# Directional DBS of the Fornix in Alzheimer’s Disease Achieves Long-Term Benefits: A Case Report

**DOI:** 10.3389/fnagi.2022.809972

**Published:** 2022-04-01

**Authors:** Juan A. Barcia, María Aurora Viloria, Raquel Yubero, Leyre Sanchez-Sanchez-Rojas, Amanda López, Bryan Andrew Strange, María Cabrera, Leonides Canuet, Pedro Gil, Cristina Nombela

**Affiliations:** ^1^Departamento de Cirugía, Facultad de Medicina, Servicio de Neurocirugía, Hospital Clínico San Carlos, Universidad Complutense de Madrid, IdISSC, Madrid, Spain; ^2^Departamento de Medicina, Facultad de Medicina, Servicio de Geriatría, Hospital Clínico San Carlos, Universidad Complutense de Madrid, IdISSC, Madrid, Spain; ^3^Departamento de Neurología, Hospital Quirón Salud, Madrid, Spain; ^4^Universidad Pontificia de Comillas, Madrid, Spain; ^5^Grupo de Investigación en Neurociencias Aplicadas, Hospital Clínico San Carlos, IdISSC, Madrid, Spain; ^6^Departamento de Medicamentos Veterinarios, Agencia Española del Medicamento y Producto Sanitario, Madrid, Spain; ^7^Unidad de Investigación y Ensayos Clínicos, Instituto de Investigación Sanitaria Hospital Clínico San Carlos, Madrid, Spain; ^8^Facultad de Salud, Universidad Internacional de la Rioja, Logroño, Spain; ^9^Laboratory for Clinical Neuroscience, Centro de Tecnología Biomédica, Universidad Politécnica de Madrid, Campus de Montegancedo, Madrid, Spain; ^10^Servicio de Medicina Nuclear, Hospital Clínico San Carlos, IdISSC, Madrid, Spain; ^11^Departamento de Neurología, Hospital Nuestra Señora del Rosario, Madrid, Spain; ^12^Departamento de Psicología Biológica y de la Salud, Facultad de Psicología, Universidad Autónoma de Madrid, Madrid, Spain

**Keywords:** Alzheimer’s disease, directional deep brain stimulation, fornix (brain), clinical trial, neuropsychology

## Abstract

**Background:**

Current treatments for Alzheimer’s disease (AD) modulate global neurotransmission but are neither specific nor anatomically directed. Tailored stimulation of target nuclei will increase treatment efficacy while reducing side effects. We report the results of the first directional deep brain stimulation (dDBS) surgery and treatment of a patient with AD in an attempt to slow the progression of the disease in a woman with multi-domain, amnestic cognitive status.

**Methods:**

We aimed to assess the safety of dDBS in patients with AD using the fornix as stimulation target (primary objective) and the clinical impact of the stimulation (secondary objective). In a registered clinical trial, a female patient aged 81 years with a 2-year history of cognitive decline and diagnoses of AD underwent a bilateral dDBS surgery targeting the fornix. Stimulation parameters were set between 3.9 and 7.5 mA, 90 μs, 130 Hz for 24 months, controlling stimulation effects by 18F-fluoro-2-deoxy-D-glucose (18F-FDG) scans (baseline, 12 and 24 months), magnetoencephalography (MEG) and clinical/neuropsychological assessment (baseline, 6, 12, 18, and 24 months).

**Results:**

There were no important complications related to the procedure. In general terms, the patient showed cognitive fluctuations over the period, related to attention and executive function patterns, with no meaningful changes in any other cognitive functions, as is shown in the clinical dementia rating scale (CDR = 1) scores over the 24 months. Such stability in neuropsychological scores corresponds to the stability of the brain metabolic function, seen in PET scans. The MEG studies described low functional connectivity at baseline and a subsequent increase in the number of significant connections, mainly in the theta band, at 12 months.

**Conclusion:**

The dDBS stimulation in the fornix seems to be a safe treatment for patients in the first stage of AD. Effects on cognition seem to be mild to moderate during the first months of stimulation and return to baseline levels after 24 months, except for verbal fluency.

**Clinical Trial Registration:**

[https://clinicaltrials.gov/ct2/show/NCT03290274], identifier [NCT03290274].

## Introduction

According to the WHO, approximately 50 million people have Alzheimer’s disease (AD) or other dementias worldwide, and this figure is expected to increase three times by 2050 (Hebert et al., 2003; Brookmeyer et al., 2011). AD is a neurodegenerative disorder with cortical and subcortical features, altering the circuits involved in cognitive functions such as memory and attention, among others. Until now, advances in early diagnosis and pharmacological treatment have not led to a significant change in the prognosis of AD (Jakobs et al., 2020). Current therapies modulate neurotransmission and alleviate some symptoms, increasing the quality of life of patients and relatives, but they do not really modify the course of the disease. The tendency to study personalised treatments aims to provide anatomically directed therapies (De Matteis et al., 2018). Therefore, it is necessary to propose alternative approaches, such as deep brain stimulation (DBS), a technique that allows us to modulate the activity of the affected areas to normalise their function. This technique has shown a greater efficacy and safety in other disorders typical of elderly patients, such as Parkinson’s disease.

Just a few nuclei may work as candidates for DBS stimulation, one of which is the fornix (Senova et al., 2020). The fornix is a voluminous axonal tract that constitutes the main route of entry and exit of information to the hippocampus and the medial temporal lobe (Jakobs et al., 2020). In 2010, a phase I trial targeted the fornix in 6 patients with mild AD. After a 1-year follow-up, there were improvements in glucose metabolism levels and a slowdown in the progression of general cognitive impairment, memory in particular (Laxton et al., 2010). A later study observed stability at the cognitive level and enhanced glucose metabolism in the mesial temporal area after 12 months of stimulation in the fornix in a patient with mild cognitive impairment (Fontaine et al., 2013; Fontaine and Santucci, 2021). Another phase II clinical trial (*n* = 42 patients) demonstrated the safety of the treatment. The results indicated that efficacy varied with age: patients under 65 had a marked worsening after 1 year of stimulation. In contrast, those over 65 years of age showed a slight improvement in the cognitive sphere (Lozano et al., 2016). Similar results appeared in a later study (Targum et al., 2021).

From the methodological point of view, technical advances provide significant improvements in DBS. Although advanced stimulation offered a real-time adaptation of the stimulation provided, directional DBS (dDBS) allows more accuracy. The radially segmented design of directional electrodes reduces side effects by personalising the stimulation area: delivering either a perpendicular field or a shaped one using anodes and cathodes to steer stimulation in a particular direction (Liu et al., 2020). The use of directional electrodes targetting the fornix and rechargeable batteries in patients with AD has not yet been reported.

This study aimed to assess the safety of dDBS stimulation in the fornix in a patient with mild AD (primary objective) and (secondary objective) to assess the effects of the treatment. For the latter, we evaluated the changes at metabolic, functional, and neuropsychological levels for 24 months.

This case study is part of a clinical trial designed to assess the safety and test the effect of dDBS stimulation in either fornix or the nucleus basalis of Meynert (NBM). The case reported corresponds to patient 1, who underwent a fornix dDBS surgery. There were no other patients involved in the study.

## Materials and Methods

The patient was selected in the context of a clinical trial to assess the effect of deep brain stimulation in Alzheimer s disease Estimulación Cerebral Profunda en Enfermedad de Alzheimer (ECP-EA) at the Hospital Clínico San Carlos (Madrid, Spain). Inclusion criteria for this clinical trial were (i) age between 50 and 80 years; (ii) AD diagnosis (Dubois et al., 2016) established by a specialist within the past 12 months; (iii) positive amyloid PET scan; (iv) Mini-Mental State Exam (MMSE) score between 20 and 26 points inclusive; (v) clinical dementia rating (CDR) scale score of 1; (vi) use of cholinesterase inhibitors or memantine for 1 year at least, and deterioration in the MMSE and Alzheimer’s Disease Assessment Scale-Cognitive Subscale (ADAS-Cog) scores; (vii) absence of delusions, hallucinations or other psychiatric disorders, no neurological findings, no significant concurrent medical illness, including hypertension (rated as moderate or severe) and no history of stroke or cerebral vascular disease; and (viii) patient willing and able to provide consent. The study had approval from the Hospital Clínico San Carlos Ethics Committee (code: ECP-EA - CI 13/448). The study is registered at clinicaltrials.gov under the trial name “Clinical Trial to Evaluate the Efficacy and Security of Deep Brain Stimulation in Alzheimer’s Disease (ECP-EA),” registration number: NCT03290274 (https://clinicaltrials.gov/ct2/show/NCT03290274).

### Case Presentation

A female patient aged 81 years diagnosed with AD joined the NCT03290274–ECP-EA clinical trial in 2017. She was diagnosed with AD 1 year earlier (2016), presenting recent memory loss, space disorientation, lack of attention, and difficulties in decision-making.

The participant was right-handed and had 8 years of official education. Her medical history was notable for high blood pressure, deep vein thrombosis in the lower-left limb, hiatal hernia, and cataract surgery. After diagnosis, the participant started a regimen of donepezil 10 mg, omeprazole 20 mg, losartan 50 mg, and simvastatin 40 mg. Family history was negative for AD.

At the medical history screening visit, a review was conducted of all previous diagnostic tests, neuropsychological assessments, and specific assessments for dementia (see below). On that visit, the patient signed the informed consent form and received a copy. The baseline assessments (1 week before surgery) included a physical examination, vital signs (blood pressure, pulse, and temperature), and complete blood count with differential and comprehensive metabolic panel, electrocardiogram, chest X-ray, and neuroimaging scans, such as magnetoencephalography (MEG), magnetic resonance imaging (MRI) with diffusion tensor imaging, 18F-fluoro-2-deoxy-D-glucose PET (18F-FDG-PET) and amyloid-β PET scans. The electrocardiogram showed sinus rhythm (70 bpm) and isolated supraventricular extrasystoles. Brain MRI indicated moderate cerebral and temporal medial atrophy, and amyloid-β PET was positive for amyloid deposits. The MEG study showed a somewhat disconnected brain, dominated mainly by few, relatively weak, connections, especially in both temporal regions. All data were compatible with AD diagnoses.

### Assessment Procedures

The patient underwent a specific assessment for AD, using the MMSE (Folstein et al., 1975), the CDR (Morris, 1997), and the ADAS-Cog (Rosen et al., 1984) to evaluate the cognitive changes over time.

The patient also underwent an extensive neuropsychological assessment to explore her cognitive performance, including verbal memory, working memory, processing speed, and executive and attentional functions. The neuropsychological assessment included the Word List Test from the Wechsler Memory Scale (WMS) (Weschler, 2008), Direct and Reverse Digit Span, Symbol Digit from WAIS-IV (Weschler, 2008), Trail Making Test Parts A (TMT-A) and B (TMT-B) (Reitan and Wolfson, 1993), and Phonological Verbal Fluencies from the Controlled Oral Word Association Test (COWAT) (Loonstra et al., 2001) and Semantic Verbal Fluency (Benton, 1968). Scores for the neuropsychological assessment at baseline and along the two-years follow up is available in Table 1. Before surgery, structural and functional data were acquired on a 3T Siemens TRIO system (Siemens, Erlangen, Germany). A standard eight-channel head coil was used to acquire MPRAGE T1-weighted anatomical images with 1 mm^3^ resolution [repetition time (TR), 2,300 ms; echo time (TE), 2.98 ms; flip angle, 9°] and diffusion-weighted images. White matter connectivity was quantified using probabilistic tractography with FDT Probtrackx 2.0 (Behrens et al., 2007).

**TABLE 1:** Neuropsychological and clinical scores during the study.

		Pre-Surgery		Follow-up		
			
TESTS	Subscales	Time 0	6 months	12 months	18 months	24 months
ADAS-Cog	Word Recall Task	17	17	16	18	17
	Following Commands	0	0	0	4	0
	Constructional Praxis	3	2	2	1	1
	DelayWordRecal	7	10	10	10	9
	Naming Objects	0	0	0	0	0
	Ideational Praxis	2	1	2	1	2
	Orientation	2	2	6	3	7
	Word Recognition	4	10	10	12	10
	Remembering Direc	0	1	0	0	0
	Spoken Language	0	0	0	0	0
	Word-Finding Difficulty	1	2	2	1	0
	Comprehension	0	0	0	0	1
	Concentration	1	0	1	1	0
	TOTAL	37	45	49	51	47
	TOTAL	37	45	49	51	47
Maze ADAS-Cog	Secs	14	9	5	6	20
	Error	0	0	0	0	0
Cancelation ADAS-Cog	Correct	20	22	16	15	18
	Error	0	0	0	0	0
MMSE	Orientation	7	7	5	6	3
	Fixation	3	3	3	3	3
	Attention & calculation	1	5	4	5	5
	Memory	2	0	0	0	0
	Language	8	9	8	7	8
	total	19	24	18	19	17
WMS	Learning	20; Pe 11	25; Pe 10	20; Pe 8	15; Pe 6	17; Pe 7
	Immediate Memory	5	3	2	0	2
	Long Term Memory	0; Pe 6	0; Pe 6	0; Pe 6	0; Pe 6	0; Pe 6
	Recognition	12; Pe 4	16; Pe 6	14; Pe 5	4; Pe 2	15; Pe 5
Phonological	Average	8	10	9	10	10
Fluency	F	10	8	9	11	10
	A	6; Pc 41-51	15; Pc 98	9; Pc 72-81	10; Pc 82-89	10; Pc 82-89
	S	9; Pc41-59	8; Pc 41-59	10; Pc 41-59	10; Pc 41-59	11; PC 90-92
Semantic		7	9	8	10	8
Fluency	Animals	6; Pc<1	9; Pc 3-5	8; Pc 2	12; Pc 11-18	n/a
	Fruits	7	9	7	10	8
Digit Span	Forwards	6; Pc 82-89	6; Pc 82-89	5; Pc 41-59	6; Pc 82-89	6; Pc 82-89
	Backwards	4; Pc 72-81	5; Pc 90-94	4; Pc 72-81	5; Pc 90-94	4; Pc 72-81
Letter-Number WAIS		3	3	3		3
Coding	Score	31; Pc 60-71	28; Pc41-59	37; Pc 72-81	n/a	n/a
	Error	0	0	0	n/a	n/a
Symbol Search	Score	17	20	17	n/a	n/a
	Error	2	0	1	n/a	n/a
TMT	A-Time	68; Pc 29-40	62; Pc 41-59	47; Pc 60-71	55; Pc 41-59	68; Pc 29-40
	A-Error	0	0	0	0	0
	B-Time	151;Pc 29-40	120;Pc41-59	208; Pc 19-28	150; Pc 29-40	95; Pc 72-81
	B-Error	1	4	4	3	7
CDR-Clinical Dementia Rating		1	1	1	1	1

*These tests were standardised in a Spanish population, and values provided stand for raw scores and percentile scores (right-hand side of the cell). For Wechsler Memory Scale (WMS), values provided stand for raw scores and standardised scores (not percentiles). “n/a” stands for missing values.*

The neuroimaging assessment also included an 18F-FDG-PET scan. PET images were generated using a PET-CT Siemens Biograph True Point platform integrating a 6-slice detector with the latest-generation PET scanner featuring a lutetium oxyorthosilicate crystal array. Static amyloid PET-CT images were acquired 90 min after intravenous injection of 216 MBq of 18F-florbetaben. Acquisition time was 15 min. The patient fasted a minimum of 6 h before the 18-FDG-PET scans. FDG (mean dose: 185 MBq) was administered intravenously 30 min before images were taken. During this time, the patient rested in a dark room with her eyes closed. Glucose levels had been checked previously and found to be lower than 150 mg/dl.

**FIGURE 1:**
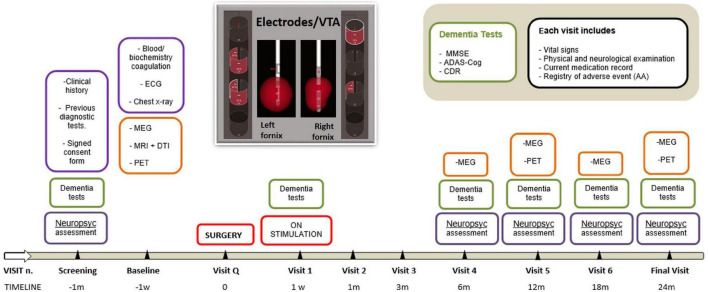
The timeline of the study.

Magnetoencephalography was conducted using a 306-channel Elekta Neuromag^®^ (Elekta Oy, Helsinki, Finland). The data ranging from 1 to 45 Hz were filtered by removing ocular and cardiac artefacts were removed by the independent component analysis (ICA) method. The signal was decomposed into functionally distinct frequency bands, such as delta (1–4 Hz), theta (4–8 Hz), alpha (8–12 Hz), beta (12–30 Hz), and gamma (30–45 Hz). Contemporarily, the cortex surface was divided into 68 regions of interest (ROIs) based on the Brainstorm default atlas (Desikan et al., 2006). The time series of each ROI was extracted by taking the average of all the signals coming from the dipoles of that region. With the aim of measuring functional connectivity between all pairs of regions (68 × 68) for each frequency band (Lachaux et al., 1999), the time series of the sources were extracted with the phase-locking value (PLV) algorithm. Details of the assessment timeline appear in Figure 1.

### Therapeutic Intervention

#### Presurgery

On the day before the operation, a volumetric CT scan (Philips^®^ Brilliance 64 CT Scanner) was performed in hospital and fused with contrast-enhanced T1 MR and T2 volumetric images. The process included a 3T MRI and CT for navigation planning, presurgery. Initially, the columns of both fornices were identified and a target was selected, 2 mm anterior and parallel to the vertical portion of the fornix. The most ventral contact was located 2 mm above the dorsal surface of the optic tract, approximately 5 mm from the midline.

#### Surgery

The Vercise Cartesia Directional Leads (Boston Scientific^®^, Iberica) for dDBS were implanted under NeuroNavigator and fluoroscopic control in the awake patient. Then, intraoperative stimulation was applied to identify beneficial or adverse effects, including unpleasant sensations (sweating, hallucinations, visual sensations, or paraesthesia). Later, a subcutaneous pulse generator (M365DB12000 Vercise™ Gevia System) was implanted in the abdomen, together with subcutaneous connections from the electrodes to the generator under general anaesthesia. A brain CT scan was performed to rule out complications (pneumocephalus or bleeding) on the day of surgery. On the day after surgery, another CT scan was performed to confirm the correct location of the electrodes.

#### Postsurgery

The patient was monitored in the intensive care unit for 24 h after the operation. Subsequently, the patient was shifted to the ward and monitored for 3 days before being discharged without significant events. At 1 week after the surgery, monopolar stimulation therapy was started in the bilateral fornix at threshold intensity (90 μs, 130 Hz) to verify the presence of side effects. Then, stimulation was monopolar, the 2 negative distal electrodes and the positive casing, as follows: a) right hemisphere, 7.5 mA: contacts 2 (–30%), 4 (–30%), 5 (–20%), 7 (–20%) and housing positive (+ 100%); b) left hemisphere, 3.9 mA: contacts 4 (30%), 5 (–70%) and housing positive (+ 100%). For more details, see Figure 1.

At 1 month after discharge, the patient was admitted to neurosurgery for suspected infection from an implanted dDBS device. An urgent chest ultrasound revealed a liquid collection surrounding the implanted device but not extending beyond the chest pocket itself. The medical staff prescribed an antibiotic treatment (meropenem and línezolid). During her stay, the patient then remained clinically stable; the inflammatory signs in the area of the implanted stimulator battery progressively reduced. She completed the 2-week antibiotic cycle and was discharged 14 days later.

The medication remained unchanged throughout the trial. The same pattern of stimulation was maintained over 24 months, with visits scheduled after 1 week and 1, 3, 6, 12, 18, and 24 months from the start of stimulation (Figure 1). Subsequent protocol visits were attended by the geriatrician, neurosurgeon, and neuropsychologist.

#### Follow-Up and Outcomes

Follow-up continued in the memory unit of the geriatric service according to the ECP-EA protocol, in the absence of adverse events.

## Results

### Neuropsychology

The basal cognitive assessment of the patient showed scores below the average in most of the tasks applied: ADAS-Cog, MMSE, WMS list of words, verbal fluency, and processing speed. At 6 months after the surgery, the scores showed mild improvement in tasks related to executive and attentional patterns: TMT-A; TMT-B, digit span backward, verbal fluency, and mazes of the ADAS-Cog. No changes were observed in the patient’s performance of any other tasks. In general terms, the patient showed cognitive fluctuations over the period, related to attention and executive function patterns, with no meaningful changes in any other cognitive functions, as is shown in the CDR scores over 24 months.

### Neuroimaging Data

At baseline, the MEG study indicated few, relatively weak connections, especially between both temporal regions, whereas the PET study showed mild temporal hypometabolism. These data agreed with the moderate cortical atrophy described in the MRI scan at baseline, particularly in the medial temporal regions.

**FIGURE 2:**
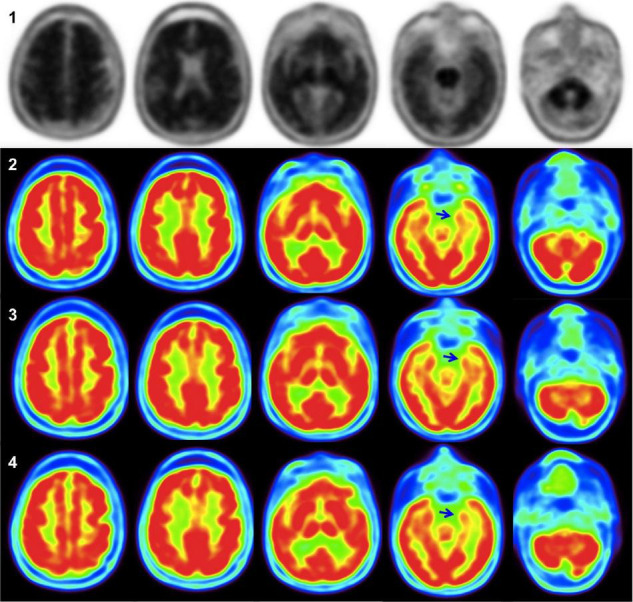
In amyloid PET images **(1)**, the uptake of the grey matter equals that of the white matter in the lateral temporal, parietal, posterior cingulum/precuneus, and especially in the frontal lobe, thus being considered a positive study for amyloid deposition. 18F-FDG-PET images at diagnosis **(2)** show only mild mesial hypometabolism, predominantly in the left hemisphere (blue arrow). 18F-FDG-PET studies showed no evolution of hypometabolism at 1 year **(3)**, and 2 years after diagnosis **(4)** (blue arrows).

The comparison between the PET studies at baseline and the 1- and 2-year follow-ups indicated no progression in the mild bilateral hypometabolism described in the mesial temporal regions at baseline (Figure 2).

Magnetoencephalography recordings were conducted at baseline, 6, 12, 18, and 24 months after surgery. Baseline MEG images were derived from the connectivity analysis between all brain areas of the first recording. This helped us to define the connectivity threshold for poststimulation comparisons. At baseline, very few areas were functionally connected or synchronised, particularly at the bilateral temporal level. After 6 months of dDBS stimulation, there was an increased number of significant connections, mainly in the theta band. In particular, we found significant changes in both interhemispheric and intrahemispheric functional connectivity affecting temporal areas, with a predominant pattern of significant hypoconnectivity. After 12 months of stimulation, we found important connectivity changes. At 18 months, the effect persisted but to a lesser degree, and at 24 months there remained a low number of connections, showing, in particular, relative hyperconnectivity, as is usually seen in the early stages of AD.

### Clinical Impression

The clinical and neuropsychological evaluation indicates that, after implantation of the electrodes in the fornix (bilateral), the patient managed to remain cognitively stable during 2 years of follow-up, showing slight cognitive and functional deterioration in the last evaluation.

The patient showed good adherence (attending appointments for clinical visits, scans, and assessments). Both patient and relatives verbally declared satisfaction with the treatment and the effects. No subsequent diagnostic or complications were reported.

Finally, on the last visit of the ECP-EA clinical trial, the patient presented Katz B (urinary incontinence), requiring help for all instrumental activities but walking without additional support.

## Discussion

Alzheimer’s disease is an increasingly prevalent, serious social and health problem worldwide. Current medication provides relief from symptoms, but disease progression remains intact. This study describes the first dDBS intervention in a patient with AD by stimulating the fornix in both hemispheres. After completing the 24-month follow-up according to the protocol, we can conclude that dDBS of the fornix constitutes a safe procedure, without serious side effects and well-tolerated, in agreement with a previous study. Regarding the efficacy of this therapeutic option, the cognitive and functional stability of the patient was observed during the whole study. The stimulation provided a transient cognitive conversion at the neuropsychological and functional levels (as assessed by PET and MEG), by increasing brain metabolism and neural connectivity in the temporal brain hubs.

**FIGURE 3:**
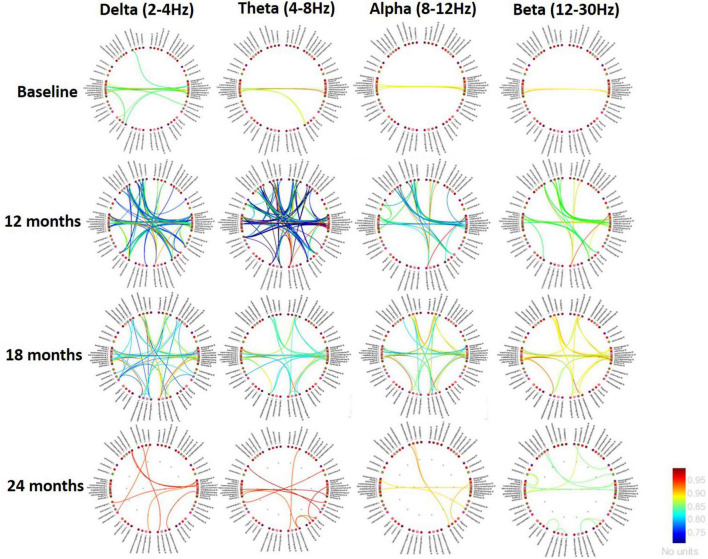
Changes in brain connectivity from the initial study to the end of follow-up (24 months). Red and yellow lines indicate an increase in connectivity, while the blue lines show a decreasing pattern. Functional connections predominate in the bilateral mid-temporal regions with cingulate areas and, to a lesser extent, with frontomedial and orbitofrontal areas. In other words, the connectivity between the temporal and limbic areas remains quite strong. Low frequencies show an earlier decrease in connectivity, but the latest studies would indicate a pattern of reorganisation of activity.

Regarding the MEG findings, hypersynchronisation in the theta and other frequency bands has been reported in association with a negative impact on cognition in early AD and with tau/amyloid-β pathology (Canuet et al., 2012, 2015). Thus, this evidence supports the pattern of stimulation-induced theta hypoconnectivity underlying early cognitive improvements in this patient with AD (Figure 3).

This clinical case formed part of a dDBS clinical trial intended to test its efficacy in two targets: the fornix (main input and output data mainstream of the hippocampus and media temporal lobe) and the NBM. The stimulation of the NBM responded to the hypothesis of reduced acetylcholine level at the cortical level in patients with AD. By random assignment, the first patient enrolled successfully underwent the fornix stimulation. Previous studies reported moderate beneficial effects of both fornix and NBM in AD. Specifically, DBS of NBM showed moderate improvements in cognitive measures in advanced patients (Zhang et al., 2021) and metabolic changes (Kuhn et al., 2015). Studies on evoked potential techniques showed benefits on auditory processing, linking DBS to normalisation of the P50 component under stimulation (vs. sham) (Dürschmid et al., 2020). Some additional effects have been observed in the counteraction of nutritional status deterioration and progressive weight loss (Noreik et al., 2015), together with abnormal thermoregulation (Zhang et al., 2021). In the case of fornix stimulation, relevant clinical trials have verified safety, but efficacy remains elusive. In general, the effect of fornix stimulation ranges from a reduction in the rate of cognitive decline to some possible improvements (Laxton et al., 2010; Fontaine et al., 2013; Leoutsakos et al., 2019). These changes correlated with increases in the glucose metabolism of the temporal and parietal lobes (Laxton et al., 2010) and hippocampal size (Sankar et al., 2015). One of the variables that may explain the differences among studies is the age or the status of the patients and the treatment being more beneficial in those aged over 65 (Lozano et al., 2016; Leoutsakos et al., 2019; Targum et al., 2021).

Although most of the studies used similar instruments to assess the changes related to brain stimulation, the suitability of these instruments needs to be considered. The MMSE and ADAS-Cog were designed specifically for AD and were supposed to detect changes in the progression of each case. Interestingly, opposite results are shown in the scores obtained in MMSE and ADAS-Cog for the effect of stimulation: the increase of 10 points in ADAS-Cog from the first evaluation to the last evaluation (50th percentile) contrasts with the 2-point decrease in MMSE (percentile < 1). This disparity shows that the two tests measure quite different things (Wouters, 2010). In the presymptomatic stages of the disease, the changes are slight and difficult to detect (Snyder et al., 2014), which must be considered when applying these techniques in the early stages of the disease. In this study, we consider the relevance of using a variety of scales, in addition to ADAS-Cog and MMSE, to allow the detection of subtle changes. The effect of the stimulation seems to depend, in fact, on the duration of the therapy. At 6 months, the effects relate essentially to the memory sphere, while at 12 months, an improvement in executive functions begins. Longer studies will be needed if we are to verify the stability in semantic fluency and executive functions.

The majority of DBS studies in AD (either fornix, NBM, or ventral capsule stimulation) include a follow-up period of 6–12 months (Laxton et al., 2010; Fontaine et al., 2013; Kuhn et al., 2015; Lozano et al., 2016; Scharre et al., 2018; Targum et al., 2021). In our study, four post-surgery assessments were conducted during a 24-month follow-up period, as in the study of Leoutsakos (Leoutsakos et al., 2019). Although some variations occurred in the neuropsychological scores, the general instruments reported no changes, mimicking baseline scores. Our results support the possibility that the effect of the stimulation has a peak at 12 months, which means that the follow-up period of future studies should perhaps also be 24 months. In addition, the maintenance of the baseline cognitive performance after 24 months is significant, contrary to our previous understanding of the standard progression of this disorder. These data also outline the range of possible effects linked to dDBS. They will help to establish a frame of reference for planning future studies and to state realistic objectives in cognitive rehabilitation.

One of the main limitations of the study was the difficulty in recruiting. In general terms, patients do not identify DBS as a current treatment for AD, and therefore they reject it (Fontaine et al., 2013). If future research is to assess the eventual benefits of this therapy more reliably in patients with AD, it will need to use a representative sample.

The range of therapeutic options for AD is as wide and complex as its etiopathogenesis. In the future, researchers must focus on those options that offer the highest levels of selectivity and therapeutic potential and provide the best balance of risks and benefits. It is becoming increasingly important to develop multidimensional treatments, ones that are neither focussed on a single strategy nor exclusively based on pharmacological treatment. They must be combined, translationally, with physiotherapy, neuropsychology, and occupational training (Elmaleh et al., 2019), in attempting to slow down the progression of the disease.

## Data Availability Statement

The original contributions presented in the study are included in the article, further inquiries can be directed to the corresponding author.

## Ethics Statement

The study involving human participants were reviewed and approved by the Hospital Clínico San Carlos Ethics Committee (code: ECP-EA – C.I. 13/448). The patient provided her written informed consent to participate in this study and for the publication of any potentially identifiable images or data included in this article.

## Author Contributions

JB was involved in the conceptualisation and design of the study, interpretation of results and supervision. MV, CN, AL, and LS-S-R were involved in conceptualisation and revising. MC and LC were responsible for neuroimaging acquisition, analysis and results. RY and MV were involved in data acquisition, analysis, and interpretations of results. CN was in charge of the manuscript drafting. JB, MV, CN, RY, MC, LC, AL, BS, PG, and LS-S-R were involved in the critical revision of the manuscript.

## Conflict of Interest

The authors declare that the research was conducted in the absence of any commercial or financial relationships that could be construed as a potential conflict of interest.

## Publisher’s Note

All claims expressed in this article are solely those of the authors and do not necessarily represent those of their affiliated organizations, or those of the publisher, the editors and the reviewers. Any product that may be evaluated in this article, or claim that may be made by its manufacturer, is not guaranteed or endorsed by the publisher.
